# 

**Published:** 1884-11

**Authors:** 


### CONTENTS.

COMMUNICATIONS.

Dentistry in Austria........ 529
Hysterics and Neurasthenia Caused by Dental Irritation........ 532
On the Removal of Naso-Pharyngeal Tumors........ 534
Local Anesthesia........ 537

PROCEEDINGS.

Mad River Valley Dental Society........ 539
Minnesota State Dental Society........ 544

CORRESPONDENCE.

The Robinson Remedy........ 550

SELECTIONS.

Experiments in Modelling Composition........ 552
Minnesota Medical Practice Act........ 557
On the Action of a Secretion Obtained from the Medical Leech........ 558
Chloroform Syncope Treated by Reversing........ 559
Neuralgia Caused by Exostosis........ 560
Scientific Fallacies........ 561
Lemon Juice in Malaria........ 562
The Men who are Promoted........ 562
A Test for the Purity of Idoform........ 563
Medical Education........ 564
Dental Caries........ 564
Cinnamon Bark for Tooth-Ache........ 565
Borax and Iodide of Potash for the Voice........ 565
How to Prepare a 1 to 1,000 Solution of Corrosive Sublimate........ 566
Membrane of Egg for Skin Grafting........ 566
To Cure Stammering........ 569
The Value of Vaccination........ 569
Salmagundi........ 570

EDITORIAL.

Law........ 573
The New Journal of Dentistry........ 575
The Ninth Meeting of the International Congress........ 576
Bibliographical........ 577
North Western Dental Association........ 579
Obituary........ 579
A Case........ 579

